# Kinesin KIF3A regulates meiotic progression and spindle assembly in oocyte meiosis

**DOI:** 10.1007/s00018-024-05213-3

**Published:** 2024-04-08

**Authors:** Jing-Cai Liu, Zhen-Nan Pan, Jia-Qian Ju, Yuan-Jing Zou, Meng-Hao Pan, Yue Wang, Xin Wu, Shao-Chen Sun

**Affiliations:** 1 College of Animal Science and Technology, Nanjing Agricultural University, Nanjing, China; 2 State Key Laboratory of Reproductive Medicine, Nanjing Medical University, Nanjing, China

**Keywords:** Kinesin, Oocyte, Meiosis, Cell cycle, Spindle

## Abstract

Kinesin family member 3A (KIF3A) is a microtubule-oriented motor protein that belongs to the kinesin-2 family for regulating intracellular transport and microtubule movement. In this study, we characterized the critical roles of KIF3A during mouse oocyte meiosis. We found that KIF3A associated with microtubules during meiosis and depletion of KIF3A resulted in oocyte maturation defects. LC–MS data indicated that KIF3A associated with cell cycle regulation, cytoskeleton, mitochondrial function and intracellular transport-related molecules. Depletion of KIF3A activated the spindle assembly checkpoint, leading to metaphase I arrest of the first meiosis. In addition, KIF3A depletion caused aberrant spindle pole organization based on its association with KIFC1 to regulate expression and polar localization of NuMA and γ-tubulin; and KIF3A knockdown also reduced microtubule stability due to the altered microtubule deacetylation by histone deacetylase 6 (HDAC6). Exogenous *Kif3a* mRNA supplementation rescued the maturation defects caused by KIF3A depletion. Moreover, KIF3A was also essential for the distribution and function of mitochondria, Golgi apparatus and endoplasmic reticulum in oocytes. Conditional knockout of epithelial splicing regulatory protein 1 (ESRP1) disrupted the expression and localization of KIF3A in oocytes. Overall, our results suggest that KIF3A regulates cell cycle progression, spindle assembly and organelle distribution during mouse oocyte meiosis.

**Supplementary Information:**

The online version contains supplementary material available at 10.1007/s00018-024-05213-3.

## Introduction

Molecular motors are a class of protein macromolecules that provide power for the transport of intracellular substances [1]. A subset of microtubule-based molecular motors, named kinesins, are mainly involved in the transport of vesicles, organelles, mRNAs, and proteins [2]. Kinesins play important roles in spindle formation, centromere-microtubule connection, spindle pole assembly, kinetochore-microtubule connection, and chromosome movement [3–5]. Kinesin family member 3A (KIF3A) is a subunit of hetero-trimeric kinesin 2, a microtubule plus-end-directed motor protein [6], and is a key factor in the formation and maintenance of cilia [7, 8]. Deletion of KIF3A results in defective osteoblast function and osteopenia, resulting in decreased bone mineral density [9]. In addition, KIF3A plays a crucial role in animal spermatogenesis [10]. In mice, structural deletion of KIF3A results in severe disturbance of mesoderm development, which is associated with defects in hedgehog signaling and mesoembryonic lethality [11, 12]. An in vitro study using MDCK cells found that KIF3A directs microtubule dynamics as well as cell migration and lumen formation [7]. Other studies have also shown that KIF3A controls the dynamics and orientation of microtubules. Indeed, KIF3A deficiency can lead to disordered microtubule polarity, which prevents the vertical growth of microtubules to the leading edge of cells [7, 13].

Successful completion of oocyte meiotic maturation is the basis for the production of healthy embryos. Unlike that of somatic cells, maturation of oocytes is characterized by asymmetric meiotic division, which results in the production of a large oocyte and a small polar body [14]. The retention of maternal components during the growth phase of oocytes is essential to support development after fertilization [15]. Oocyte meiosis consists of two rounds of meiotic arrest and resumption. The first round occurs in the germinal vesicle (GV) stage; upon stimulation by luteinizing hormone, the prophase I-arrested oocytes undergo germinal vesicle breakdown (GVBD) and resume the first meiosis. The second round is the block in metaphase II (MII), which is released when fertilization occurs. The maturation-promoting factors cyclin B1 and cyclin-dependent kinase 1 (CDK1) are responsible for regulating meiotic resumption and development, and an increase in the activity of CDK1 is the key to meiotic resumption [16].

The lack of a centrosome structure in oocytes means that formation of the spindle pole is not organized by a single entity; rather, spindle pole assembly is achieved by the self-organization of several microtubule-organizing centers (MTOCs) [17]. In mouse oocytes, MTOCs promote microtubule nucleation and assembly to form bipolar spindles, and the orderly arrangement of chromosomes on the equatorial plate is a achieved through the traction of kinetochore microtubules [18, 19]. The spindle migrates along the long axis to the oocyte cortex in a microfilament-dependent manner, and then extrudes the first polar body and the oocytes are arrested in MII stage [18, 20]. Formation of the spindle pole, the region where the minus ends of microtubules aggregate, is dependent on the activity of microtubule motors and microtubule-associated proteins. The nuclear mitotic apparatus (NuMA) protein is enriched at the minus-end of microtubules, where it recruits the motor protein dynein and can cross-link microtubules itself [21]. These activities allow NuMA to aggregate the minus ends of microtubules, thereby organizing spindle poles in the absence of centrosomes or MTOCs [22]. During this process, tubulin acetylation is important for regulating and maintaining the structure and integrity of microtubules, and it is mainly controlled by histone deacetylase 6 (HDAC6) and α-tubulin acetyltransferase 1 [23]. It is generally believed that the acetylation modification of microtubules is related to their stability, and that acetylation occurs for the stabilization of microtubules [24, 25].

Proper distribution and functioning of organelles are critical for mammalian oocyte maturation during meiosis [26]. In particular, the functions of mitochondria are affected by their redistribution and changes in activity that occur during the transition of oocytes from the GV to the MII stage [27, 28]. Impaired oocyte quality is associated with abnormal mitochondrial rearrangement and low ATP levels [29], and previous studies have shown that microtubules/microfilaments are involved in regulating the dynamic changes of mitochondria in oocytes [27, 30]. Mitochondria gather around the spindle via a dynamin-mediated mechanism and migrate to the cortex with the spindle, and mitochondrial aggregation is accompanied by the formation of long cortical microfilaments [31, 32]. The arrangement of the endoplasmic reticulum (ER) also changes dynamically during oocyte maturation; specifically, it is spread throughout the cytoplasm in the GV stage, but is arranged on the developing meiotic spindle in the MI stage after GVBD. This rearrangement of the ER can be prevented by inhibition of microtubule polymerization or cytoplasmic dynamin [33]. Dynamic changes in the distribution of the Golgi apparatus also occur during oocyte maturation; specifically, the Golgi is located in the cortex and nucleus at the GV stage, but migrates from the periphery to the central cytoplasmic region during the transition from GVBD to MI [34]. The structure and function of the Golgi are controlled by the actin and microtubule cytoskeletons, as well as by the minus-end dynein motor [35]. Previous studies have established that actin and actin-binding proteins regulate several events associated with the Golgi complex, such as protein sorting and membrane fission [36–38].

Although KIF3A plays an important role in regulating organelles and microtubules during mitosis, its role in oocyte meiosis is still unclear. In this study, we found that KIF3A is essential for proper cell cycle progression and meiotic spindle pole assembly in mouse oocytes, highlighting the multiple roles of kinesins in meiosis.

## Materials and methods

### Animals and oocyte culture

The 5–6-week-old ICR female mice were obtained from Nanjing Medical University (Nanjing, China). The conditional knock out mice of ESRP1 gene produced by Cre/loxP system were the gift from Prof. Xin Wu of Nanjing Medical University [39]. This study was approved by the Animal Management and Ethics Committee of Nanjing Agricultural University (NJAU.No20210402040). The mice were handled according to the guidelines of the Animal Management and Ethics Committee of Nanjing Agricultural University. A total of 332 mice were used in this study. For oocyte culture, GV stage oocytes were obtained from ovaries and then cultured in mineral oil-covered M2 (EasyCheck, M1250, Nanjing, China) medium in an incubator at 37 °C and 5% CO_2_. The oocyte stage was defined by the status of chromosome alignment and spindle morphology, together with culture time.

### Myc-KIF3A plasmid construction and mRNA synthesis

Myc-KIF3A plasmid was constructed by Wuhan GeneCreate Biological Engineering Co., Ltd. 50 μL bacteria solution with Myc-KIF3A plasmid was added to 20 mL LB medium with ampicillin, and the bacteria were shaken at 37 °C for 18 h. After the bacterial solution was collected by centrifugation at 12,000 rpm, plasmid extraction was carried out using PurePlasmid Mini Kit (Cwbio, CW0500, Wuhan, China). The plasmid linearization was then performed using AvrII. That was, plasmid 20 μg, AvrII restriction endonuclease 2 μL, cut smart 20 μL, adding RNase-free water to make the total volume 200 μL. The reaction condition was 37 °C, 2 h. mRNA synthesis was performed using m7G(5′)ppp(5′)G (New England Biolabs, S1404S) and HiScribe T7 high-yield RNA synthesis kit (New England Biolabs, E2040S, MA, USA). The reaction system was: Template DNA 1 μg, 10X Reaction Buffer 2 µL, ATP (100 mM) 2 µL, GTP (20 mM) 2 µL, CTP (100 mM) 2 µL, UTP (100 mM) 2 µL, Cap Analog (40 mM) 4 µL, T7 RNA Polymerase Mix 2 µL, plus RNase-free water for a total volume of 20 µL. The reaction condition was 37 °C, 2 h. Then the RNA Clean & Concentrato-25 (Zymo Research, R1017, CA, USA) kit was used for purification, which was to add 200 μL of RNA Binding Buffer to the solution obtained in the previous step and mix it well. Add 300 μL of anhydrous ethanol and mix gently. The mixture was transferred to a ZIMO-SPIN IICR Column adsorption column fitted with a collection tube and centrifuged at 12,000 rpm for 1 min. 400 μL RNA Prep Buffer was added into the adsorption column, centrifuged at 12,000 rpm for 1 min, and the waste solution was discarded. 700 μL RNA Wash Buffer was added into the adsorption column, centrifuged at 12,000 rpm for 1 min, and the waste liquid was discarded. 400 μL RNA Wash Buffer was added into the centrifugal column, centrifuged at 12,000 rpm for 1 min, and the waste liquid was discarded. The adsorption column was transferred to an RNase-free centrifuge tube, and 14 μL RNasefree water was added to the center of the adsorption column and left for 1 min at room temperature. The myc-KIF3A mRNA obtained by centrifugation at 13,500 rpm for 2 min was packaged and stored at −80 ℃.

### Microinjection of Kif3a siRNA and mRNA

For KIF3A knockdown, microinjection of 5–10 pL siRNA per GV stage oocyte were performed using an Eppendorf FemtoJet under an inverted microscope (Olympus IX71), and then the oocytes were cultured in M2 medium containing 50 μM IBMX for 22 h at GV stage. The oocytes were then washed eight times and transferred to fresh M2 medium for culture. The Kif3a-siRNA: F, 5′-GCA ACG UUA UUU CUG CCU UTT-3′; 5′-AAG GCA GAA AUA ACG UUG CTT-3′ (GenePharma, Shanghai, China) was diluted with water to 50 μM. An equal amount of negative control siRNA F, 5′-UUC UCC GAA CGU GUC ACG UTT-3′; 5′-ACG UGA CAC GUU CGG AGA ATT-3′ (GenePharma, Shanghai, China) without homology to mammalian genes was used as control. After 20 h siRNA microinjection, 5–10 pL Myc-KIF3A mRNA was microinjected into oocytes for rescue experiment. GV oocytes were cultured in M2 medium containing IBMX for 2 h, washed in fresh M2 medium for eight times, and then transferred to fresh M2 medium for culture. We defined the transition time of oocytes from IBMX-containing culture to IBMX-free culture as 0 h. After 4 h of oocyte culture, the germinal vesicle breakdown (GVBD) occurred. The spindle-pulled chromosomes were arranged neatly on the equatorial plate at 8 h, which was the MI stage. At 10 h, the spindle-pulled chromosomes reached the cortical area and the cytokinesis was performed at anaphase-telophase I (ATI) stage. At 12 h, the oocytes completed the first polar body extrusion, which was MII stage. We selected MI (8 h) stage oocytes for spindle morphology and organelle detection. The percentage of PB1 was the number of MII oocytes compared to the total number of oocytes after GVBD. Each subsequent analysis was performed at least 3 biological replicates.

### Taxol and nocodazole treatment of oocytes

For taxol treatment, GV (0 h) and MI (8 h) stage oocytes were transferred to M2 medium containing 10 μM taxol and incubated for 45 min. Oocytes were then fixed for subsequent staining. For nocodazole treatment, MI (8 h) oocytes were transferred to an M2 medium containing 20 μg/mL nocodazole for 10 min. Oocytes were then fixed for subsequent staining experiments. At least 30 oocytes were examined in each group, and at least three independent experiments were performed.

### Immunofluorescence staining and intensity analysis

For immunofluorescence staining, oocytes were fixed with 4% paraformaldehyde for 30 min at room temperature, then permeabilized with 0.5% Triton X-100 in PBS for 20 min, followed by incubation in PBS with 1% BSA to block 1 h. Oocytes were incubated with the identified specific primary antibodies (Table S1) for 12 h at 4 °C and then with the corresponding secondary antibodies for 1 h at room temperature. After washing three times with washing solution (0.1% Tween 20 and 0.01% Triton X-100 in PBS), oocytes were incubated with Hoechst 33,342 for 10 min, then transferred to slides for mounting. Single-layer section scanning and photographing were performed using a confocal laser scanning microscope (Zeiss, LSM 900 META). We defined the spindles with thick middle and thin poles as normal spindles; the spindles with scattered, diffuse, depolaris, or multipolar phenomena as abnormal spindles; those with neatly arranged chromosomes on the equatorial plate as normal chromosomes; and those with diffuse distribution as abnormal chromosome distribution. ImageJ was used to calculate the average fluorescence intensity of ac-tubulin, KIFC1 and KIF3A on the spindle showing with the ratio of the total fluorescence intensity of ac-tubulin, KIFC1 and KIF3A on the spindle area. The fluorescence intensity of GM130 was obtained by the ratio of the total fluorescence intensity of GM130 in the region around the spindle area. Each subsequent analysis was performed at least 3 biological replicates.

### Immunocytochemistry staining

For immunocytochemistry staining, oocytes were permeabilized with 0.5% Triton X-100 in PBS for 10 min, then fixed with 4% paraformaldehyde for 30 min at room temperature. After that, the oocytes were incubated with 3% H_2_O_2_ for 10 min, followed by the incubation in PBS with 1% BSA to block 1 h. Oocytes were incubated with the identified specific NuMA antibodies for 12 h at 4 °C and then labeled with the corresponding HRP-labeled Goat Anti-Rabbit IgG(H + L) antibodies for 1 h at room temperature. After washing three times with washing solution (0.1% Tween 20 and 0.01% Triton X-100 in PBS), chromogenic reaction was performed with DAB peroxidase substrate (ZSGB‐BIO, ZLI‐9017). After three times of washing, oocytes were transferred to slides for mounting, images were taken under a microscope (CKX53; Olympus).

### Co-immunoprecipitation and liquid chromatography mass spectrometry

Co-immunoprecipitation (Co-IP) was followed to the Dynabeads Protein G kit (ThermoFisher Scientific, 10003D) protocol. Briefly, 6 mouse ovaries were added to lysis buffer containing protease inhibitor cocktail for mechanical disruption. Rabbit anti-KIF3A antibody was incubated with Dynabeads Protein G for 1 h at room temperature. After washing three times, the antibody-conjugated dynabeads were incubated with cell lysates for 6 h at 4 °C. The tube was placed on a magnet and the bead-antibody-antigen complexes were washed three times. Then 30 μL of eluation buffer was added to elute the bead-antibody-antigen complex, and the sample was used for liquid chromatography mass spectrometry (LC–MS) or subsequent detection. LC–MS was processed and analyzed by Beijing Genomics Institute (BGI). Firstly, the obtained raw MS data was converted into MS peak file, then the mascot software was used to search and match the species sequences in the database, and the search results were filtered and quality controlled to give reliable protein identification results. The original data of LC–MS have been deposited to the ProteomeXchange Consortium via the PRIDE [40] partner repository with the dataset identifier PXD050190. All identified proteins were BLAST compared to the KOG database to obtain the corresponding KOG annotation results. STRING analysis was performed online at https://cn.string-db.org/.

### Western blotting

For western blotting, 10 μL of NuPAGE LDS Sample Buffer (Invitrogen, NP0007) was added per 160 or 180 oocytes, and then placed at 70 °C for 5 min. Samples were electrophoresed with 10% NuPAGE Novex (Invitrogen, NP0302BOX), then transferred to polyvinylidene fluoride membranes. Membranes were blocked with 5% non-fat milk in TBST for 4 h, and then incubated with the identified specific primary antibodies (Table S1) for 12 h at 4 °C. Membranes were washed three times in TBST and incubated with HRP-labeled goat goat anti-rabbit IgG (1:1000) (Beyotime, A0208) or goat anti-mouse IgG (1:1000) (Beyotime, A0216) in TBST for 90 min. Tanon High-sig ECL Western Blotting Substrate (Tanon, 180–501) was used for signal chemiluminescence and AlphaView SA software (Proteinsimple, CA, USA) was used for quantitative grayscale analysis. The control group was set to 1 to normalize the data. Three independent repeat experiments were performed.

### Mitochondria and ER detection

We performed mitochondria, ER (endoplasmic reticulum) and Golgi detection assays in MI stage oocytes of control and KIF3A-KD (knock down) groups. The oocytes were placed in the M2 medium with Mito-tracker probes (Invitrogen, M7512) and the mitochondrial potential probe 5,5′,6,6′-tetrachloro-1,1′,3,3′-tetraethylbenzimidazolyl-carbocyanine iodide (JC-1, Beyotime, C2005, Nantong, China) reagent for 30 min at 37 °C to detect mitochondrial distribution and mitochondrial membrane potential, respectively. The oocytes were placed in the M2 medium of ER-Tracker Red (Beyotime, C1041) for 30 min at 37 °C to detect ER. After washing three times with M2 medium, oocytes were stained with Hoechst 33,342 for 10 min, then transferred to Nunc glass base dish (Thermo scientific, 150,680). We observed and photographed the samples with a confocal laser scanning microscope (Zeiss, LSM 900 META).

### ATP level detection

We detected ATP levels in control and KIF3A-KD group oocytes using ATP bioluminescent assay kit (Sigma, Shanghai, China). At least 30 oocytes from each group were washed three times with PBS, and then 100 μL of ATP Releasing Reagent was added and shaken on ice for 3 min, then 100 μL of working solution was added, and the mixture was added to 96 wells for chemiluminescence detection with a Luminometer (Thermo fisher). Three independent experiments were performed.

### Statistical analysis

The data were presented as mean ± SEM and all analysis were performed using GraphPad Prism 9 software. First, whether the data was normally distributed and whether the variance was homogeneous were tested. For the data from two groups, Student’s t-test or Mann–whitney test was selected. For the data from three or more groups, analysis of Ordinary one-way ANOVA or Kruskal–Wallis test was selected. The calculated percentage rate was the ratio of the target oocytes number observed in each group to the total oocyte number. At least 3 biological replicates were performed for each analysis in this study. *P* < 0.05 were considered significant differences (*P < 0.05, **P < 0.01, ***P < 0.001).

## Results

### KIF3A is essential for mouse oocyte maturation

**Fig. 1:**
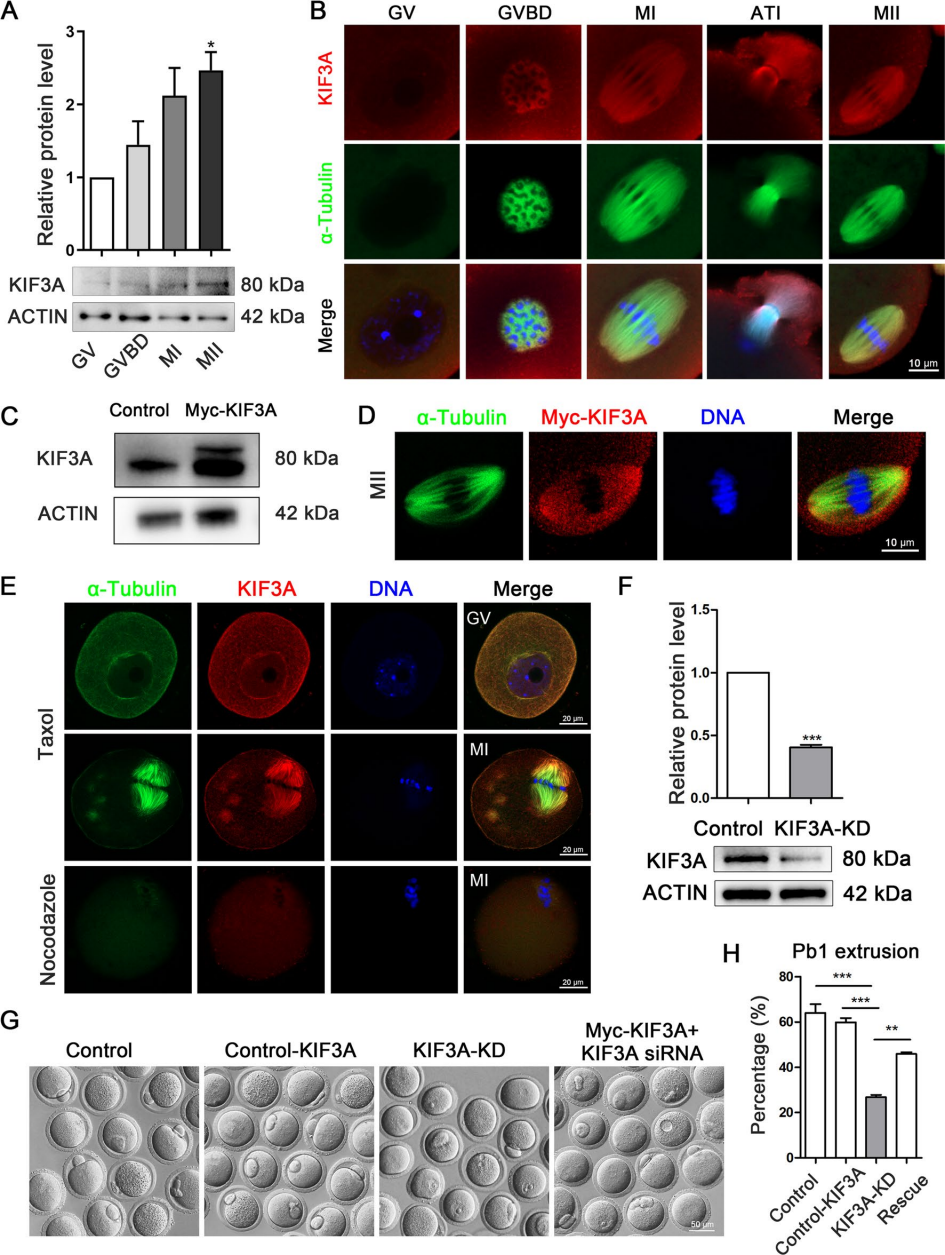
KIF3A is essential for mouse oocyte maturation. **A** Western blot analysis for KIF3A protein expression in mouse oocytes at GV (0 h), GVBD (4 h), MI (8 h) and MII (12 h) stages. 160 oocytes were used for each lane. **B** Representative images of KIF3A localization in fixed mouse oocytes at different stages. Green, α‐Tubulin; red, KIF3A; blue, Hoechst, DNA. KIF3A had no specific localization in GV stage, but localized near chromosomes after GVBD, and accumulated in meiotic spindle region in both MI and MII stages. KIF3A localized to the midbody during ATI (10 h). **C** Western blotting detection of KIF3A protein expression after microinjection of Myc-KIF3A mRNA, and two bands of KIF3A could be observed in Myc-KIF3A group. 180 oocytes (2 h after injection) were used for each lane. **D** The localization of Myc-KIF3A protein in fixed oocytes stained by Myc at MII (12 h) stage after Myc-KIF3A mRNA injection. Myc-KIF3A accumulated in meiotic spindle region. Red, Myc-KIF3A; blue, Hoechst, DNA. **E** Subcellular localization of KIF3A following taxol and nocodazole treatment at the GV and MI stage of fixed mouse oocytes. Red, KIF3A; green, α-Tubulin; blue, Hoechst, DNA. **F** Western blotting detection of KIF3A protein expression after microinjection of *Kif3a* siRNA. 180 MI (8 h) stage oocytes were used for each lane. **G** Typical images of the first polar body of oocytes cultured for 12 h between different groups. **H** The extrusion rate of the first polar body of oocytes cultured for 12 h in control (n = 126), control-KIF3A (n = 136), KIF3A-KD (n = 90) and rescue (n = 78) groups. All the control group was microinjected with an equal amount of negative control RNAs; Results are presented as mean ± SEM; experiments were repeated at least 3 times (*P < 0.05; **P < 0.01; ***P < 0.001)

First, we examined the expression and localization of the KIF3A protein during different stages of mouse oocyte meiosis. KIF3A was expressed at the GV, GVBD, MI, and MII stages, and its expression level increased gradually during meiotic maturation (Fig. 1A). An immunofluorescence analysis by different antibody staining revealed that KIF3A aggregated around chromosomes after GVBD, whereas it was localized on spindle microtubules during MI and MII, and at the midbody during telophase I (n = 71, Figs. 1B, S1).

Next, we prepared an exogenous Myc-*Kif3a* mRNA and microinjected it into oocytes to further verify its localization. Western blotting confirmed successful translation of the exogenous mRNA (Fig. 1C), and the subcellular localization pattern of Myc-KIF3A in oocytes was similar to that of native KIF3A (Fig. 1D). Subsequently, we treated oocytes in the GV or MI stage with taxol or nocodazole to promote microtubule polymerization or depolymerization, respectively, and examined the effects on KIF3A localization. KIF3A co-localized with microtubules and was enriched at asters and spindle after taxol treatment, whereas it was dispersed in the cytoplasm after nocodazole treatment (Fig. 1E).

To explore its role in mouse oocyte meiosis further, we used a specific siRNA to knock down (KD) KIF3A. Microinjection of mouse oocytes with the *Kif3a* siRNA reduced the KIF3A protein level significantly (control vs. KIF3A-KD: 1.0 vs. 0.41 ± 0.02;* P* < 0.001; Fig. 1F). The role of KIF3A in extrusion of the first polar body was then examined via microinjection of oocytes with the *Kif3a* siRNA, with or without simultaneous supplementation of exogenous Myc-*Kif3a* mRNA (Fig. 1G). We found that the first polar body extrusion rate was significantly reduced after KIF3A knockdown, and expression of the Myc-*Kif3a* mRNA rescued polar body extrusion defects caused by *Kif3a* siRNA injection (control vs. Myc-Kif3a vs. KIF3A-KD vs. KIF3A-KD + Myc-Kif3a: 63.99 ± 3.84%, n = 126 vs. 59.87 ± 1.83%, n = 136 vs. 26.75 ± 1.03%, n = 90 vs. 45.83 ± 0.80%, n = 78; Fig. 1H). Overall, these findings suggest that KIF3A plays a critical role in mouse oocyte meiotic maturation.

### KIF3A depletion causes metaphase-to-anaphase transition failure by activating SAC

**Fig. 2:**
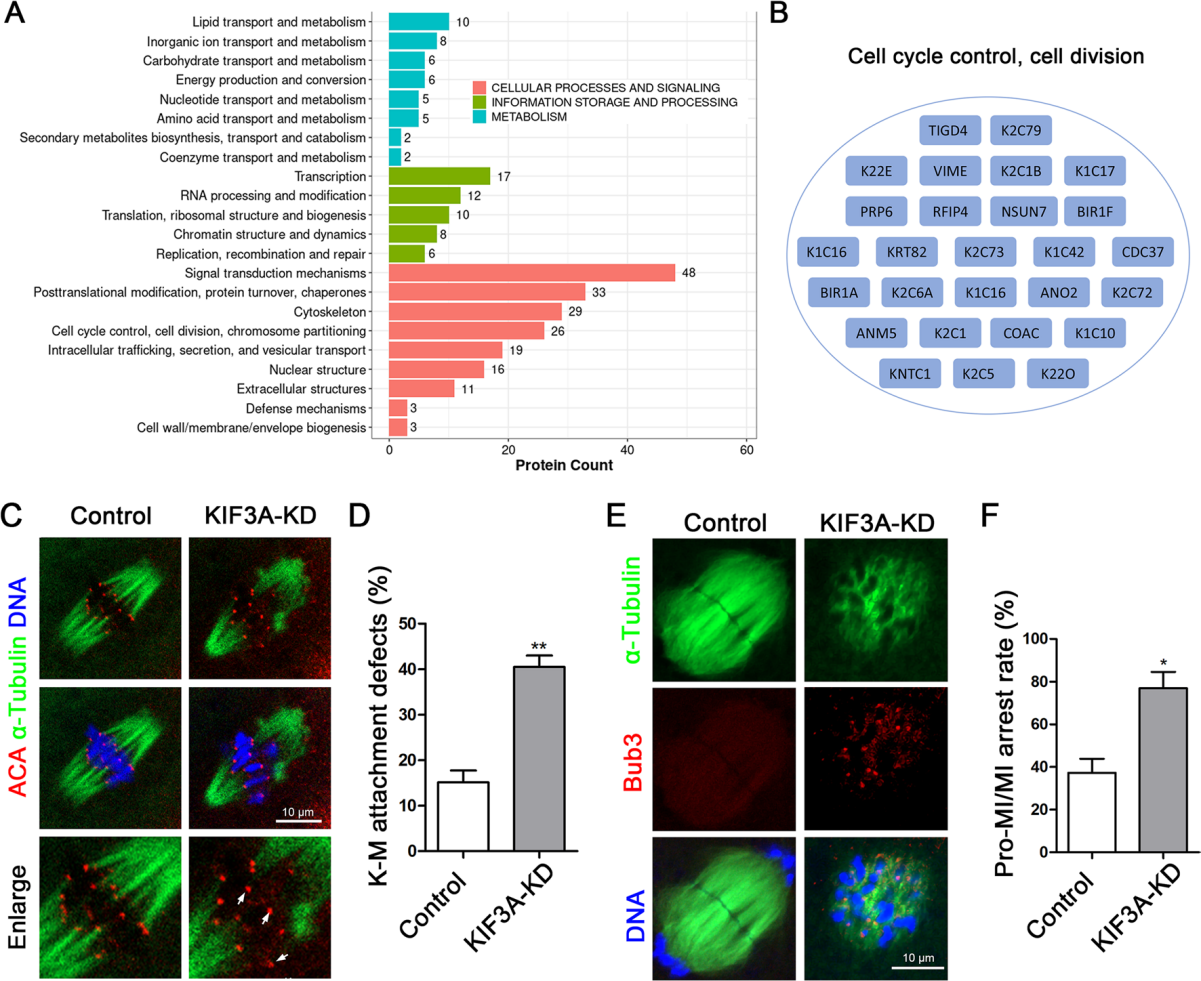
KIF3A affects cell cycle progression in mouse oocytes. **A** KOG annotation map of KIF3A-related proteins screened by LC–MS in mouse ovaries. **B** Cell cycle control, cell division related proteins in LC–MS results. **C** Typical images of ACA in fixed MI stage oocytes in the control and KIF3A-KD groups. Red, ACA; green, α-Tubulin; blue, Hoechst, DNA. **D** The rate of kinetochore-microtubule attachment defects in the control and KIF3A-KD groups. **E** Typical images of Bub3 in the oocytes fixed after 10 h of culture in the control and KIF3A-KD groups. Red, Bub3; green, α-Tubulin; blue, Hoechst, DNA. **F** The rate of Pro-MI/MI in the control and the KIF3A-KD groups after 10 h of culture. All the control group was microinjected with an equal amount of negative control RNAs; Results are presented as mean ± SEM; experiments were repeated at least 3 times (*P < 0.05; **P < 0.01)

In order to explore the cause for the defect of polar body extrusion by KIF3A knockdown, we performed co-immunoprecipitation (Co-IP) combined with LC–MS analyses to identify proteins that interact with KIF3A in mouse ovarian cells. An enrichment analysis revealed that KIF3A-related proteins were associated with cell cycle control, cell division, the cytoskeleton, nuclear structure, mitochondria, energy production, intracellular trafficking, secretion, and vesicular transport (Fig. 2A). We found that KIF3A potentially associated with several cell cycle control and cell division-related proteins such as CDC37, BIR1A etc. (Fig. 2B). Further analysis showed that depletion of KIF3A disturbed microtubule-kinetochore attachment (Fig. 2C) and a large proportion of oocytes expressing the siRNA harbored microtubules that were not connected to kinetochores (control vs. KIF3A-KD: 15.13 ± 2.60%, n = 33 vs. 40.47 ± 2.51%, n = 43; *P* < 0.01; Fig. 2D). This caused the failure of metaphase I to anaphase I transition, which might be due to the Bub3-based activation of the spindle assembly checkpoint (SAC) after KIF3A depletion. Indeed, in line with the active Bub3 signals on kinetochores, a large proportion of oocytes expressing the KIF3A siRNA were arrested at the pro-MI or MI stage (control vs. KIF3A-KD: 37.30 ± 6.50%, n = 45 vs. 76.86 ± 7.69%, n = 39; *P* < 0.01; Fig. 2E, F), reflecting activation of the SAC. These results demonstrate that KD of KIF3A affects the progression of mouse oocyte meiosis.

### KIF3A regulates microtubule stability to control spindle organization in mouse oocytes

**Fig. 3:**
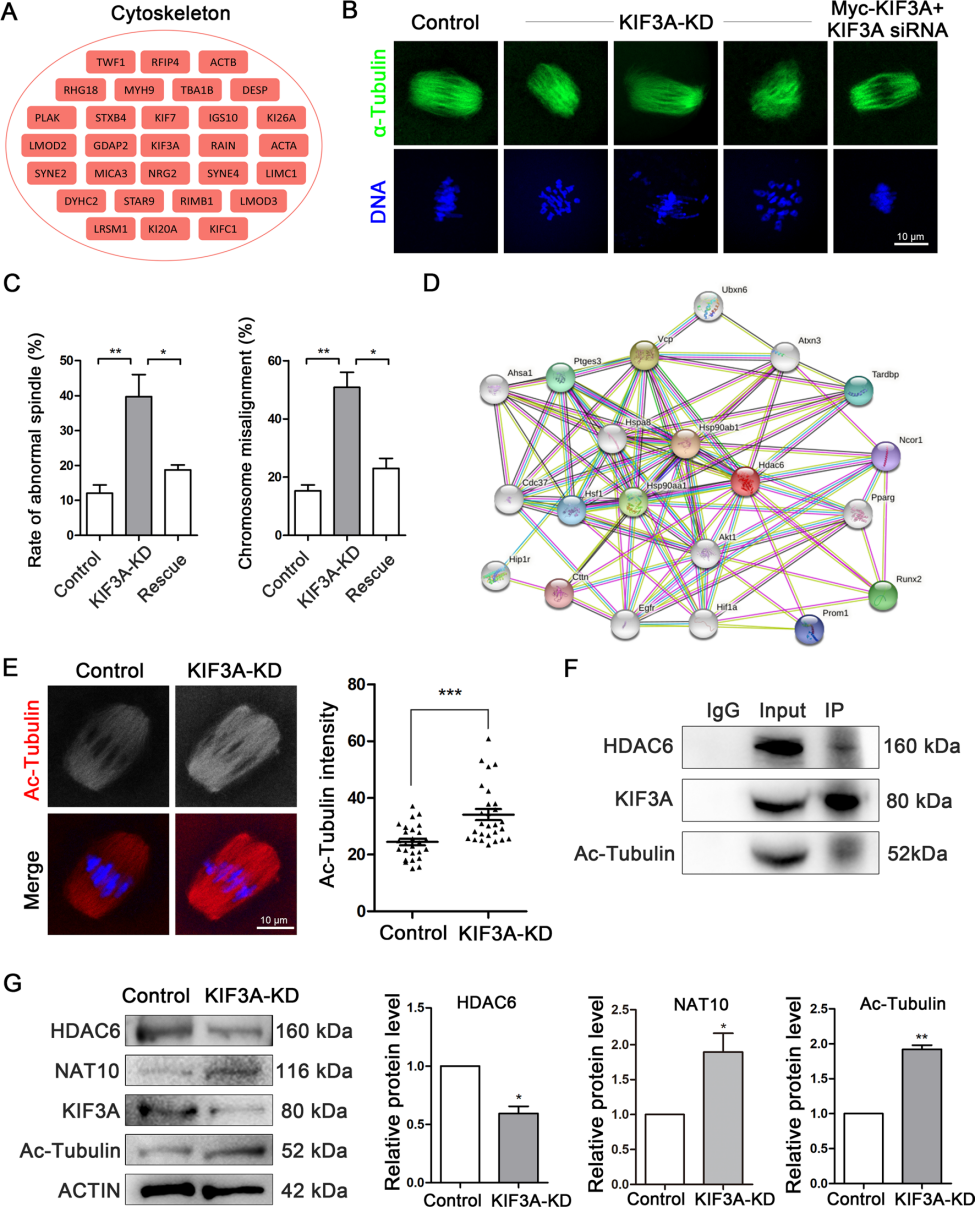
KIF3A regulates microtubule stability for spindle organization in mouse oocytes. **A** Cytoskeleton-related proteins in LC–MS results. **B** Typical images of meiotic spindle of fixed oocytes in the control, KIF3A-KD and Rescue groups. Spindle, green; DNA, Hoechst, blue. **C** The rate of abnormal spindle and chromosome misalignment in the control, KIF3A-KD and Rescue oocytes. (Abnormal spindles: control 12.05 ± 2.75%, n = 66 vs KIF3A-KD 39.68 ± 10.98%, n = 59 vs Rescue 18.76 ± 1.41%, n = 60; chromosomal misalignment: control 15.30 ± 2.02%, n = 63 vs KIF3A-KD 50.81 ± 5.21%, n = 62 vs Rescue 23.02 ± 3.50%, n = 60). **D** STRING analysis for the potential interaction proteins with KIF3A. AKT1 and CDC37 related with KIF3A from MS data might associated with HDAC6, α-tubulin deacetylase. **E** Typical images and fluorescence intensity analysis of ac-tubulin in MI stage fixed oocytes of control (n = 26) and KIF3A-KD (n = 27) groups. Red, ac-tubulin; blue, Hoechst, DNA. **F** Co-IP results showed that KIF3A associated with HDAC6 and ac-Tubulin in ovarian cells. **G** Western blotting results and intensity analysis of microtubule acetylation-related proteins of HDAC6, NAT10 and Ac-Tubulin. 180 MI (8 h) stage oocytes were used for each lane. All the control group was microinjected with an equal amount of negative control RNAs; Results are presented as mean ± SEM; experiments were repeated at least 3 times (*P < 0.05; **P < 0.01; ***P < 0.001)

The LC–MS analysis suggested that a large number of cytoskeleton-related proteins might interact with KIF3A (Fig. 3A). We examined the effect of KD of KIF3A on meiotic spindle morphology in mouse oocytes. Compared with control oocytes, KIF3A-KD oocytes had significantly higher numbers of abnormal spindles with morphological defects, including malformed and multipolar spindles, as well as a significantly higher number of seriously misaligned abnormal chromosomes, all of which could be rescued by injection of oocytes with exogenous Myc-*Kif3a* mRNA (control vs. KIF3A-KD vs. KIF3A-KD + Myc-Kif3a: abnormal spindles: 12.05 ± 2.75%, n = 66 vs. 39.68 ± 10.98%, n = 59 vs. 18.76 ± 1.41%, n = 60; chromosomal misalignment: 15.30 ± 2.02%, n = 63 vs. 50.81 ± 5.21%, n = 62 vs. 23.02 ± 3.50%, n = 60; Fig. 3B, C).

Next, we performed a STRING analysis of the MS data and found that the cell cycle-related proteins AKT1 and CDC37 might associate with the α-tubulin deacetylase HDAC6, which is thought to regulate microtubule acetylation (Fig. 3D). This finding lends support to a relationship between microtubule acetylation/stability and cell cycle progression. We also found that the mean fluorescence intensity of acetylated tubulin in KIF3A-KD oocyte spindles was significantly higher than that of acetylated tubulin in control oocyte spindles (control vs. KIF3A-KD: 24.45 ± 1.15, n = 26 vs. 34.09 ± 1.97, n = 27; *P* < 0.001; Fig. 3E). In a subsequent Co-IP analysis, HDAC6 and acetylated tubulin both interacted with KIF3A (Fig. 3F). Furthermore, western blotting revealed that the expression level of HDAC6 was decreased significantly in KIF3A-KD oocytes compared with control oocytes, whereas the levels of acetyltransferase N-acetyltransferase 10 (NAT10) and acetylated tubulin were increased significantly (Fig. 3G). Overall, these findings suggest that KIF3A affects the level of microtubule acetylation via effects on HDAC6, leading to abnormalities in mouse oocyte spindle formation.

### KIF3A associates with KIFC1 to ensure NuMA-based spindle pole organization in mouse oocytes

**Fig. 4:**
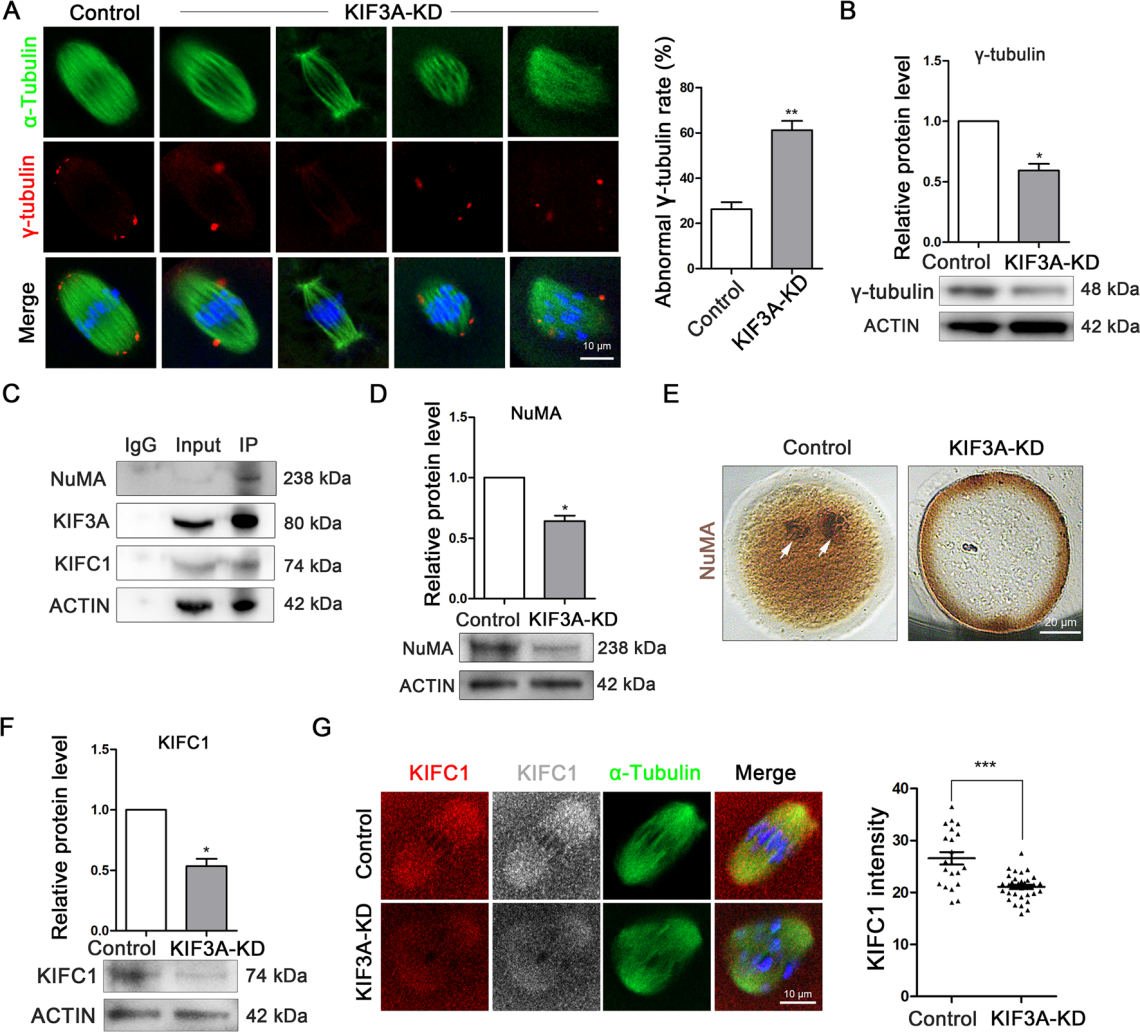
KIF3A associates with KIFC1 for NuMA-based spindle pole organization in mouse oocytes. **A** Typical images and abnormal γ-Tubulin ratio in MI (8 h) stage fixed oocytes of control (n = 39) and KIF3A-KD (n = 44) groups. Red, γ-Tubulin; green, α-Tubulin; blue, Hoechst, DNA. **B** Western blotting detection of γ-Tubulin protein expression after microinjection of *Kif3a* siRNA. 180 MI (8 h) stage oocytes were used for each lane. **C** Co-IP results showed that KIF3A associated with NuMA and KIFC1 in ovarian cells. **D** Western blotting detection of NuMA protein expression after microinjection of *Kif3a* siRNA. 180 MI (8 h) stage oocytes were used for each lane. **E** Typical images of NuMA in MI (8 h) stage fixed oocytes of control and KIF3A-KD groups. Brown, NuMA. **F** Western blotting detection of KIFC1 protein expression after microinjection of *Kif3a* siRNA. 180 MI (8 h) stage oocytes were used for each lane. **G** Typical images and fluorescence intensity statistics of KIFC1 in MI (8 h) stage fixed oocytes of control (n = 21) and KIF3A-KD (n = 33) groups. Red, KIFC1; green, α-Tubulin; blue, Hoechst, DNA. All the control group was microinjected with an equal amount of negative control RNAs; Results are presented as mean ± SEM; experiments were repeated at least 3 times (*P < 0.05; **P < 0.01; ***P < 0.001)

Labeling of γ-tubulin, a marker of MTOCs, revealed that control oocytes showed a normal state at the poles of the spindle, whereas a significant number of KIF3A-KD oocytes displayed an abnormal distribution of MTOCs, including deviation from the poles and loss of or scattered patterns (control vs. KIF3A-KD: 26.19 ± 3.12%, n = 39 vs. 61.21 ± 4.03%, n = 44; *P* < 0.01; Fig. 4A). The expression level of γ-tubulin was also reduced in the KIF3A-KD group (control vs. KIF3A-KD: 1.0 vs. 0.59 ± 0.06; Fig. 4B).

LC–MS and Co-IP analyses verified that KIF3A interacted with NuMA and KIFC1 (Fig. 4C), and western blotting revealed that the expression level of NuMA was decreased significantly in the KIF3A-KD group (control vs. KIF3A-KD: 1.0 vs. 0.64 ± 0.08; *P* < 0.05; Fig. 4D). Immunocytochemical staining of NuMA at the MI stage indicated that NuMA clustered at the spindle poles in the control group, whereas this localization was not apparent in the KIF3A-KD group (Fig. 4E). In addition, KD of KIF3A reduced the protein level of KIFC1 (control vs. KIF3A-KD: 1.0 vs. 0.53 ± 0.06; *P* < 0.05; Fig. 4F) as well as its fluorescence intensity (control vs. KIF3A-KD: 26.55 ± 3.17, n = 21 vs. 21.05 ± 1.44, n = 33; *P* < 0.001; Fig. 4G) in oocytes. Taken together, these findings indicate that KIF3A plays an essential role in spindle pole organization by interacting with KIFC1 and NuMA in mouse oocytes.

### KIF3A is essential for organelle distribution in mouse oocytes

**Fig. 5:**
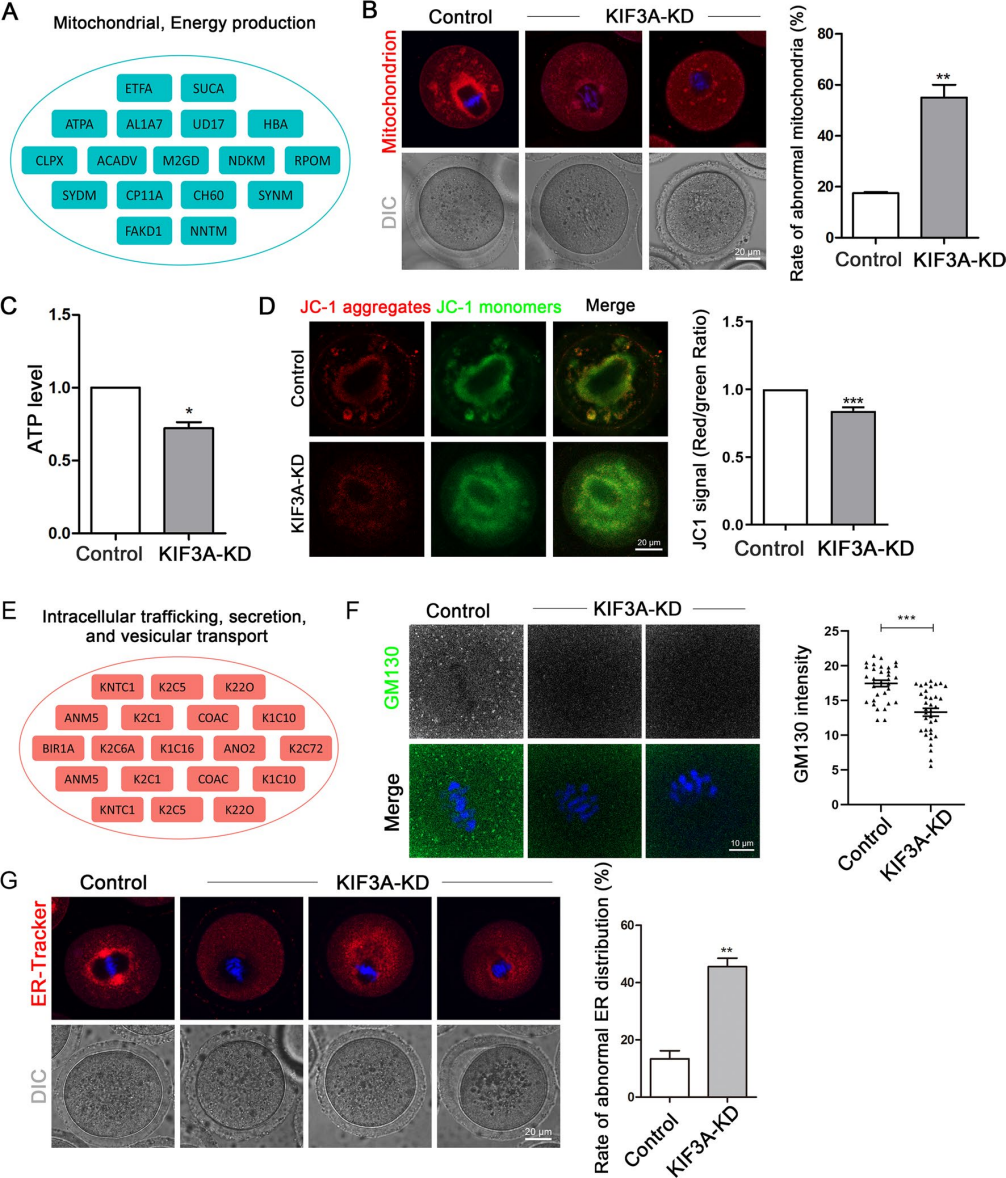
KIF3A is essential for organelle distribution in mouse oocytes. **A** Mitochondrial and energy production-related proteins in LC–MS results. **B** Typical mitochondrion images and the rate of abnormal mitochondrial in oocytes at MI (8 h) stage in the control (n = 57) and KIF3A-KD (n = 52) groups. Red, mitochondrion; blue, Hoechst, DNA. **C** Relative ATP level of MI (8 h) stage oocytes in the control (n = 90) and KIF3A-KD (n = 90) groups. **D** The typical images of mitochondrial membrane potential of JC-1 staining of oocytes at MI (8 h) stage. The ratio of JC-1 aggregates (Red) to JC-1 monomers (Green) in the KIF3A-KD (n = 41) group decreased compared with the control (n = 43) group. Red, JC-1 aggregates; Green, JC-1 monomers. **E** Intracellular trafficking, secretion and vesicular transport-related proteins in LC–MS results. **F** Typical images and fluorescence intensity statistics of GM130 in the fixed MI (8 h) stage oocytes of control (n = 32) and KIF3A-KD (n = 35) groups. Green, GM130; blue, Hoechst, DNA. **G** Typical ER images and the rate of abnormal ER in oocytes at MI (8 h) in the control (n = 40) and KIF3A-KD (n = 52) groups. Red, ER-Tracker; blue, Hoechst, DNA. All the control group was microinjected with an equal amount of negative control RNAs; Results are presented as mean ± SEM; experiments were repeated at least 3 times (*P < 0.05; **P < 0.01; ***P < 0.001)

An LC–MS enrichment analysis revealed that KIF3A-interacting proteins included a large number of mitochondria-related proteins (Fig. 5A). Mitochondria are highly dynamic during oocyte maturation; therefore, we examined the effects of KD of KIF3A on these dynamics by labeling mitochondria in MI (8 h) stage oocytes with Mito-Tracker probes. In control oocytes, mitochondria accumulated around the spindle, whereas this distribution pattern was disrupted in KIF3A-KD oocytes. In addition, compared with control oocytes, a higher proportion of KIF3A-KD oocytes displayed abnormal mitochondria (reduced number or diffuse distribution) (control vs. KIF3A-KD: 17.50 ± 0.44%, n = 57 vs. 55.01 ± 4.97%, n = 52; *P* < 0.01; Fig. 5B). Further analysis showed that the ATP level was significantly lower in the KIF3A-KD group than in the control group (control vs. KIF3A-KD: 1.0 vs. 0.72 ± 0.04; *P* < 0.05; Fig. 5C). A change in the mitochondrial membrane potential is another important indicator of mitochondrial function. We found that the mitochondrial membrane potential (the ratio of JC-1 aggregates to JC-1 monomers in the cytoplasmic region) decreased significantly after KD of KIF3A (control vs. KIF3A-KD: 1.0 vs. 0.84 ± 0.03; *P* < 0.01; Fig. 5D).

Other KIF3A-interacting proteins that were enriched in the LC–MS analysis was related to intracellular trafficking, secretion, and vesicular transport (Fig. 5E). Therefore, we examined the effects of KD of KIF3A on the Golgi apparatus by labeling the Golgi-related protein Golgi matrix protein 130 (GM130). In MI stage control oocytes, GM130 was distributed in vesicles around the spindle; however, in KIF3A-KD oocytes, this vesicle structure was diminished and the average fluorescence intensity of the Golgi region was reduced significantly (control vs. KIF3A-KD: 17.46 ± 0.46%, n = 32 vs. 13.32 ± 0.58%, n = 35; *P* < 0.001; Fig. 5F).

Next, we labeled mouse oocytes with ER-Tracker dye and found that the ER of control oocytes was concentrated around the spindle. KD of KIF3A disrupted this localization pattern and increased the proportion of oocytes displaying an abnormal ER (reduced or dispersed around the spindle) significantly (control vs. KIF3A-KD: 13.38 ± 2.85%, n = 40 vs. 45.56 ± 2.94%, n = 52; *P* < 0.01; Fig. 5G).

Overall, these findings indicate that KIF3A affects the distribution and function of the mitochondria, Golgi apparatus, and ER during oocyte meiosis.

### ESRP1 determines the expression and localization of KIF3A in mouse oocytes

**Fig. 6:**
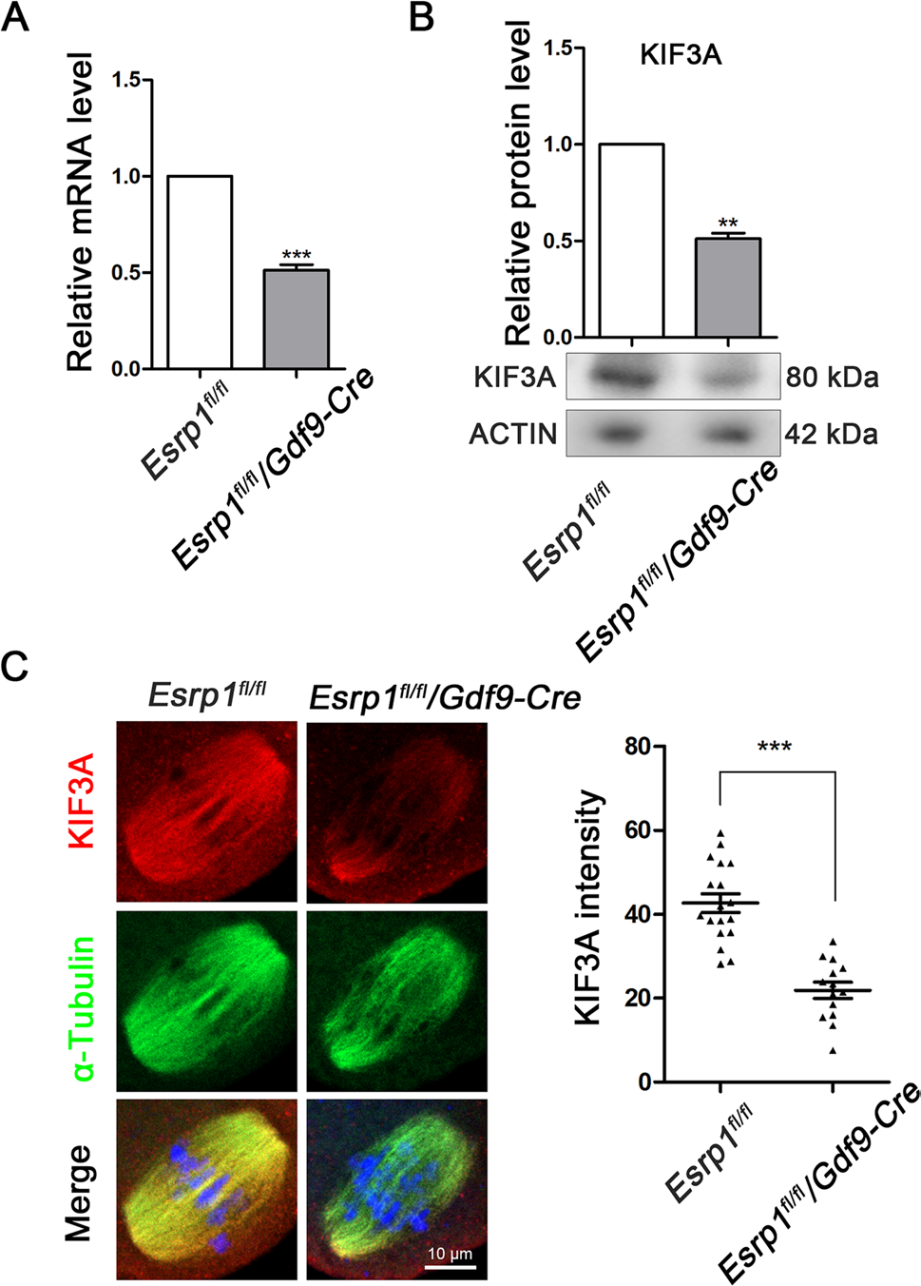
ESRP1 determines the expression and localization of KIF3A in mouse oocytes. **A** mRNA level of* Kif3a* in Esrp1^fl/fl^ and Esrp1^fl/fl^/GDF9-Cre mouse oocytes. **B** Protein level of KIF3A in Esrp1^fl/fl^ and Esrp1^fl/fl^/GDF9-Cre mouse oocytes. **C** Typical images and fluorescence intensity statistics of KIF3A in fixed MI (8 h) stage oocytes of Esrp1^fl/fl^ (n = 18) group and Esrp1^fl/fl^/GDF9-Cre (n = 14) group. Red, KIF3A; green, α-Tubulin; blue, Hoechst, DNA. Results are presented as mean ± SEM; experiments were repeated at least 3 times (**P < 0.01; ***P < 0.001)

A recent study indicated that loss of ESRP1 induced oocyte maturation defects through its effects on the gene-splicing sites of *Kif3a* and others [39]. Next, we examined the effect of knockout of epithelial splicing regulatory protein 1 (ESRP1) on KIF3A expression using donated mice with Cre/loxP-mediated conditional inactivation of the *Esrp1* gene. The expression level of the major isoform of *Kif3a* mRNA in mouse oocytes was reduced significantly after ESRP1 knockout (Fig. 6A). This change was reflected by a concomitant reduction in the KIF3A protein level (Fig. 6B) and KIF3A fluorescence staining (Fig. 6C), indicating that ESRP1 determines the expression and localization of KIF3A in mouse oocytes.

## Discussion

As a kinesin, KIF3A has been reported to play an important role in cilia formation, osteogenic development, and mitosis of various cell types [41, 42]. Previous studies have demonstrated that KIF3A is distributed mainly on spindle microtubules and centrosomes during mitosis [42]. Here, we found that it is also located on microtubules during female meiosis and is essential for the maturation of mouse oocytes.

Using LC–MS, Co-IP, KD, and exogenous mRNA rescue approaches, we found that KIF3A was essential for cell cycle regulation, meiotic spindle assembly, and organelle distribution in oocytes via effects on HDAC6-based microtubule stability and NuMA-based spindle pole formation. The LC–MS analysis revealed that KIF3A associated with multiple progress regulators related to the cell cycle, cytoskeleton, transport, and membrane organelles. Similarly, a previous study found that depletion of KIF3A and KIF3B impairs the long-distance transport of RAB5 endosomes in interphase cells, resulting in delayed nuclear membrane rupture and prolonged prophase [41]. In addition, another study found that ablation of KIF3A can reduce the cell proliferative capacity in a microtubule-dependent manner [43].

We found that KIF3A was essential for the transition of mouse oocytes from MI to anaphase I and its depletion activated the SAC. Our findings also suggest that KIF3A regulates spindle organization in mouse oocytes. Consequently, we explored the potential mechanisms underlying the effects of KIF3A on cell cycle progression and meiotic spindle formation. Previous studies have demonstrated that enhanced kinesin activity is related to tubulin acetylation [44, 45], and kinesins are thought to regulate acetylation and participate in spindle assembly in oocytes [46, 47]. Nucleolar acetyltransferase NAT10 associates with the motor protein Eg5 (KIF11) to promote formation of the mitotic spindle and maintenance of normal cell cycle progression [48]. Here, we found that the KD of KIF3A increased the expression of NAT10. In view of the synergy between NAT10 and the kinesin family, we speculate that the increase of NAT10 in this study may be a negative feedback mechanism for the down-regulation of the kinesin protein KIF3A. Moreover, we found that KIF3A knockdown reduced deacetylase HDAC6 expression, and increased the level of acetylated tubulin. In addition, KIF3A interacted with HDAC6 in mouse oocytes. Previous studies have shown that HDAC6 defects cause abnormal spindle morphology and reduced first polar body extrusion [49]. Since KIF3A is a kinesin for intracellular cargo transport, it may be involved in transporting HDAC6/SIRT2 and/or NAT10 to specific locations to regulate microtubule acetylation levels [50, 51]. When KIF3A is absent, the transport efficiency of HDAC6/SIRT2 would likely be reduced, leading to an increase in the level of NAT10 and an increase in the tubulin acetylation level. HDAC6 interacts with CDK1 [52, 53]; therefore, it is possible that, in addition to effects on tubulin acetylation and stability, the decrease in HDAC6 expression caused by KD of KIF3A may also have disturbed the CDK1 expression-related resumption of meiosis. Indeed, acetylation-related changes have been reported to affect the cell cycle, and inhibition of HDAC6 increases the level of acetylated α-tubulin, which causes a delay in the G1/S transition of the cell cycle, followed by mitotic arrest [54, 55].

Proper assembly of the bipolar spindle poles is critical for spindle organization and accurate chromosome segregation [56]. We found that after KIF3A-KD, MTOCs protein γ-tubulin was also decreased in addition to the abnormal localization. It has been reported that kinesin protein is involved in the localization process of MTOCs [22]. Our results showed that KIF3A might be involved in the formation of spindle poles, which might be induced by the abnormal localization of γ-tubulin, and since γ-tubulin cannot reach the designated position correctly, it may cause negative feedback regulation, resulting in a degradation of its protein expression. Loss of NuMA leads to spindle defects in oocyte meiosis, and KIFC1 assists NuMA in regulating the formation of spindle poles [22]. Here, we identified NuMA and KIFC1 as interacting partners of KIF3A, suggesting that these proteins coordinate to regulate spindle pole formation. Our previous studies have shown that deletion of the *Kifc1* gene affects spindle formation and thus meiosis of mouse oocytes, and another group demonstrated that KIFC1 stabilizes microtubules and collaborates with NuMA to maintain spindle pole aggregation [22]. Therefore, we conclude that KIF3A may be involved in spindle pole formation together with NuMA and KIFC1.

Organelle rearrangement is crucial for successful oocyte meiosis [57, 58] and the results presented here suggest that KIF3A affects the distribution of mitochondria, the ER, and the Golgi apparatus in mouse oocytes. Microfilaments and microtubules are essential for organelle functions [34, 59] and kinesin functionally regulates organelle dynamics [2, 60]. The regulation of organelles by microfilaments/microtubules/kinesin has been reported in oocytes [61]. Rearrangement of the ER after GVBD is dependent on microtubules and dynein dynamics, and is supported by microfilaments [62]. Contact sites between the mitochondrial outer membrane and the ER play crucial roles in mitochondrial dynamics and inheritance, membrane architecture and dynamics, metabolite/ion transport, protein and lipid biogenesis [63]. Our results suggest that KIF3A affects mitochondrial membrane potential and ER distribution, thereby weakening communication between mitochondria and ER. Abnormal functioning of organelles affects the spindle assembly; for example, the destruction of mitochondrial function in mouse oocytes leads to the abnormal formation of spindles [64]. In addition, Golgi-associated proteins regulate spindle assembly and separation during mouse oocyte meiosis [65]. Regarding the potential mechanism underlying the relationship between organelle function and spindle assembly, there is increasing evidence that the movement of organelles is influenced by microtubule acetylation [66, 67]. Increased acetylation of microtubules promotes the binding and motility of dynein and kinesin [45, 68], and it also can promote the ER sliding dynamics and ER-mitochondrial contacts [66], and results in a more dispersed mitochondrial distribution [67]. These previous findings are consistent with the organelle distributions observed in control and KIF3A-KD oocytes in our current study. In addition, other studies have shown that KIF3A is involved in the transport of mitochondria, the Golgi apparatus, and the ER [69, 70].

Depletion of ESRP1 in mouse oocytes results in maturation defects and female infertility [39]. Here, we found that loss of ESRP1 reduced KIF3A expression in mouse oocytes. The role of ESRP1 in regulating the expression of KIF3A during oocyte maturation may be related to its effects on splicing, since alternative splicing contributes to gene diversification and ESRP1 controls pre-mRNA splicing events for female fertility in mice. Indeed, deletion of ESRP1 interferes with gene splicing sites, including those of *Kif3a*, which may result in abnormal spindle organization [39]. Since mRNA and protein level were not completely reduced in the ESRP1 KO oocytes, another possibility is that Esrp1 may regulate KIF3A through its regulation on the transcription activity, which requires more evidences in the future.

## Conclusions

In conclusion, our study demonstrates that ESRP1-mediated KIF3A plays important roles in G2/M transition, meiotic spindle pole formation, metaphase-anaphase transition, and organelle rearrangement through its effects on HDAC6-mediated microtubule deacetylation and KIFC1-related NuMA during mouse oocyte meiosis.

### Supplementary Information

Below is the link to the electronic supplementary material.

Supplementary file 1 (TIF 2324 KB)

Supplementary file 2 (PNG 146 KB)

Supplementary file 3 (XLS 75 KB)

Supplementary file 4 (PNG 101 KB)

Supplementary file 5 (XLS 9 KB)

Supplementary file 6 (XLS 166 KB)

Supplementary file 7 (XLS 62 KB)

Supplementary file 8 (DOCX 18 KB)

Supplementary file 9 (DOCX 13 KB)

## Data Availability

All data generated or analyzed during this study are included in this published article and its supplementary information files. The datasets used and/or analyzed during the current study are available from the corresponding author on reasonable request.

## Abbreviations

KIF3A: Kinesin family member 3A
ESRP1: Epithelial splicing regulatory protein 1
GV: Germinal vesicle
GVBD: GV breakdown
MI: Metaphase I
ATI: Anaphase-telophase I
MII: Metaphase II
CDK1: Cyclin-dependent kinase 1
MTOCs: Microtubule-organizing centers
NuMA: Nuclear mitotic apparatus
HDAC6: Histone deacetylase 6
NAT10: N-acetyltransferase 10
JC-1: 5,5′,6,6′-Tetrachloro-1,1′,3,3′-tetraethylbenzimidazolyl-carbocyanine iodide
ER: Endoplasmic reticulum
GM130: Golgi matrix protein 130
Co-IP: Co-immunoprecipitation
SAC: Spindle assembly checkpoint
