# Supplementary figures and images for: Kinesin KIF3A regulates meiotic progression and spindle assembly in oocyte meiosis
Source: Cell Mol Life Sci. 2024 Apr 8;81(1):168. doi: 10.1007/s00018-024-05213-3 (PMC11001723; doi:10.1007/s00018-024-05213-3)

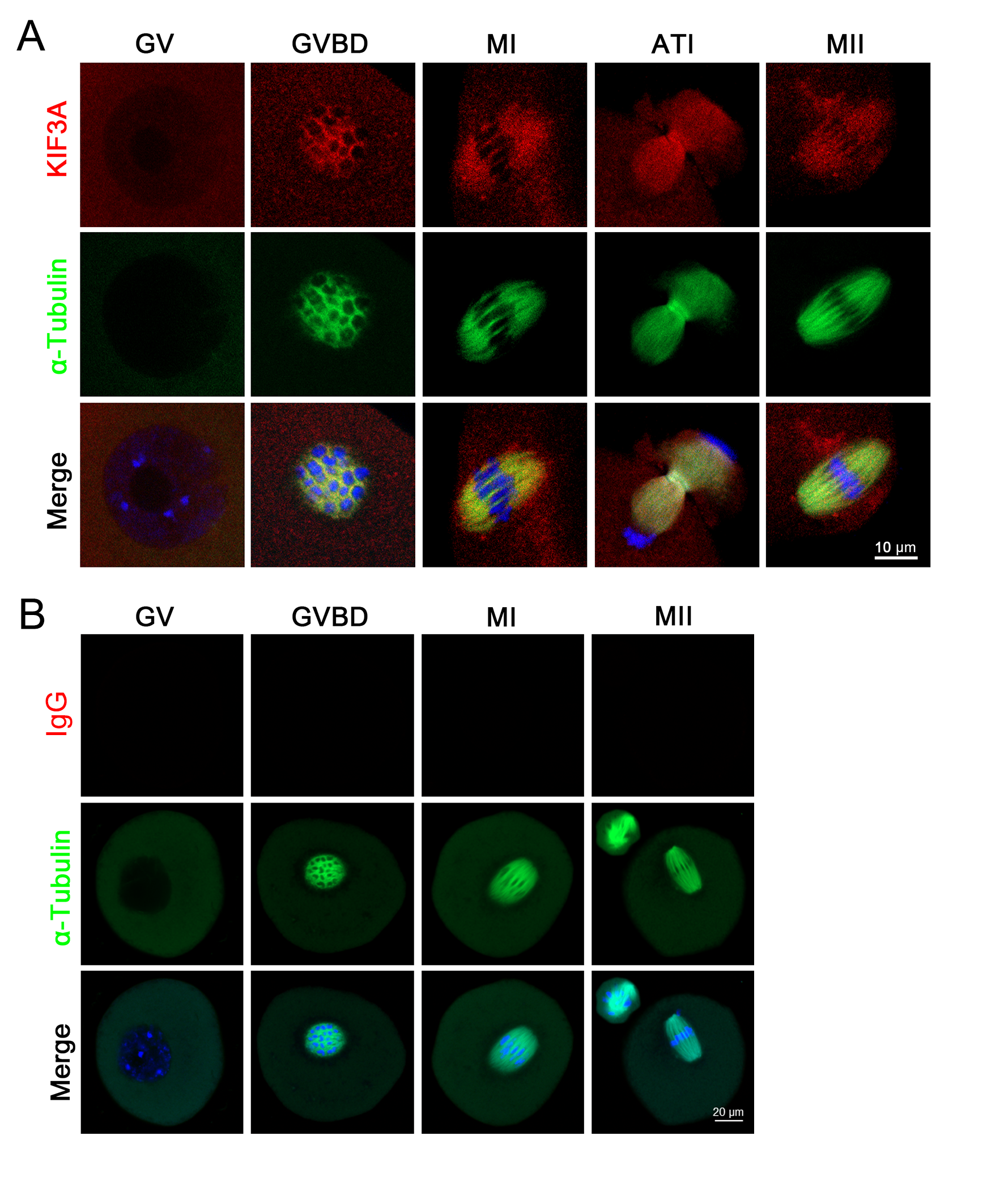

Supplement: Supplementary file 1 — (TIF 2324 KB) [file 18_2024_5213_MOESM1_ESM.tif]

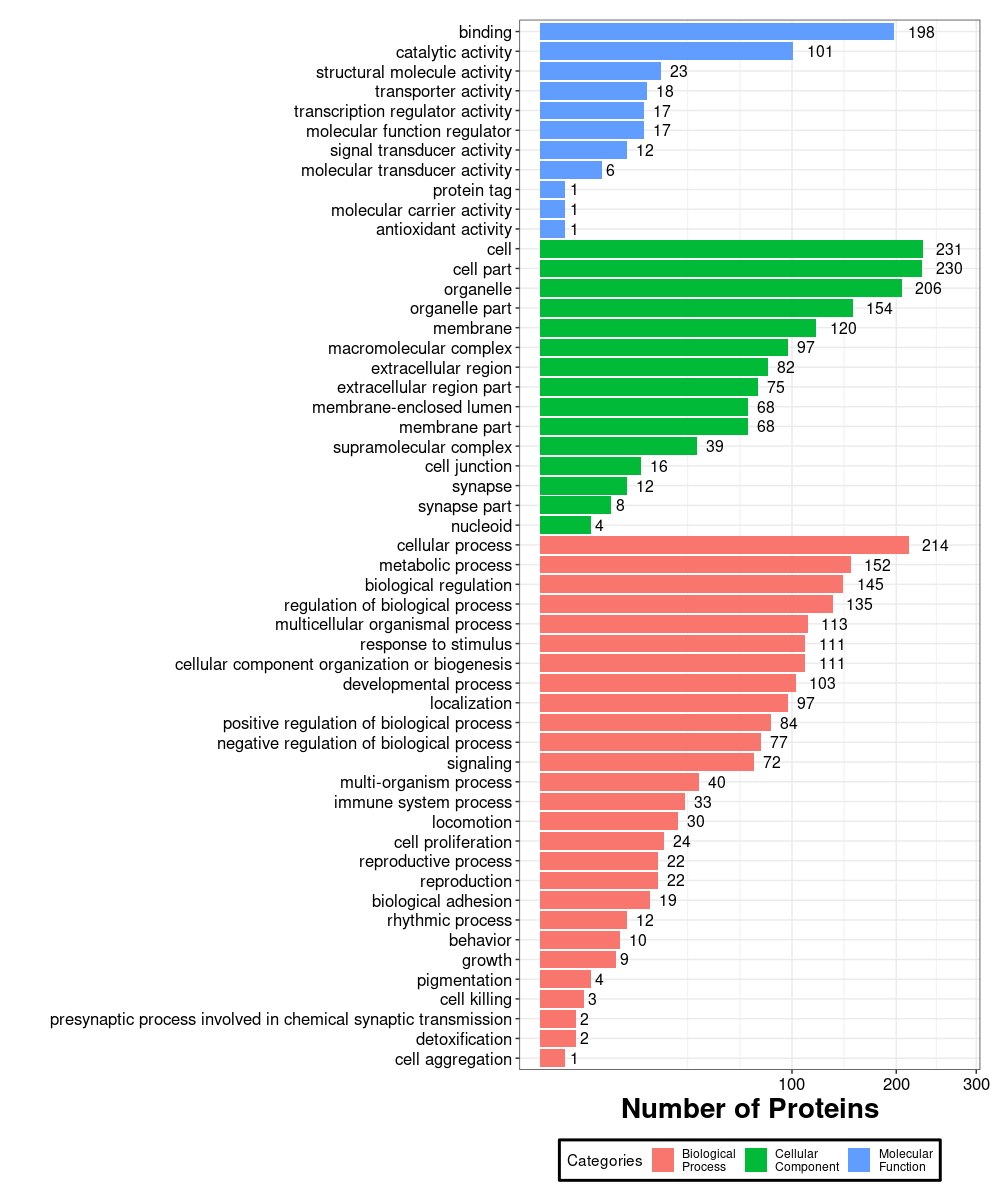

Supplement: Supplementary file 2 — (PNG 146 KB) [file 18_2024_5213_MOESM2_ESM.png]

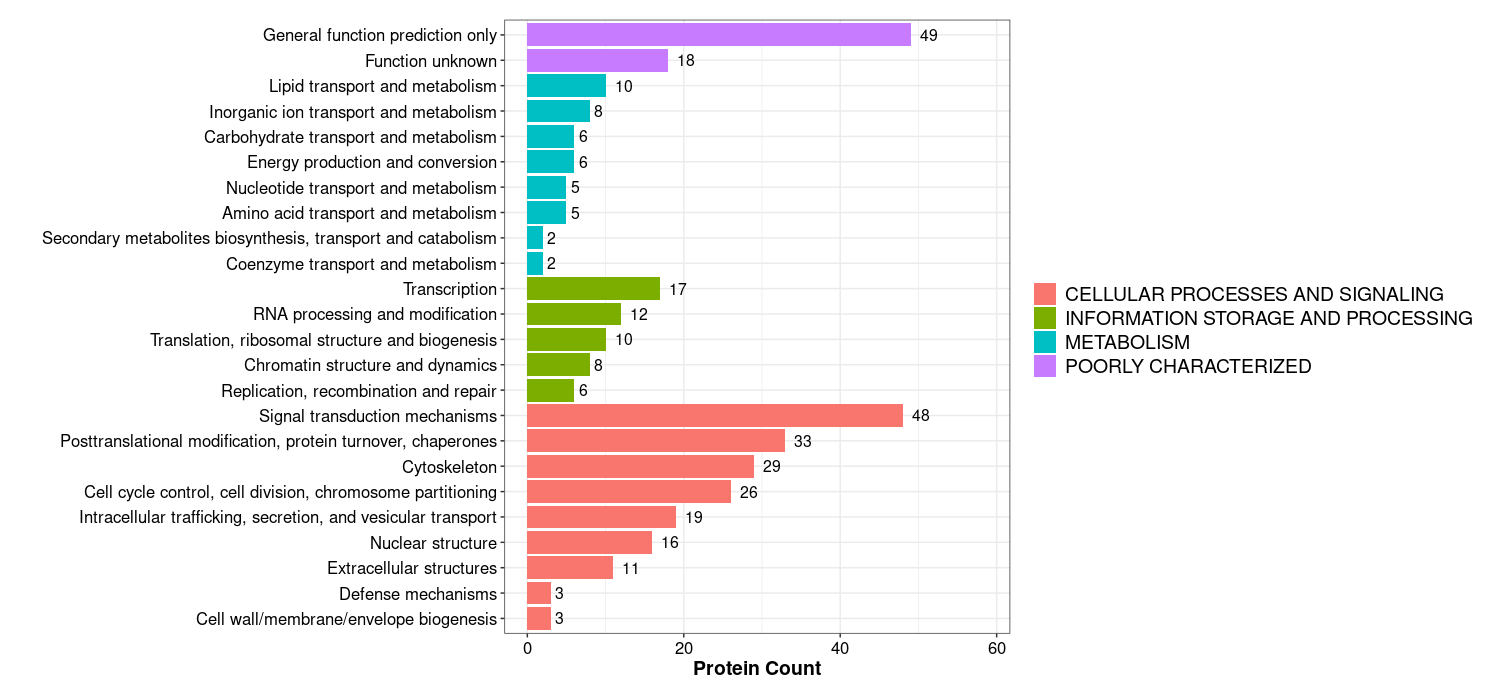

Supplement: Supplementary file 4 — (PNG 101 KB) [file 18_2024_5213_MOESM4_ESM.png]
