# Supplementary material for: Kinesin KIF3A regulates meiotic progression and spindle assembly in oocyte meiosis
Source: Cell Mol Life Sci. 2024 Apr 8;81(1):168. doi: 10.1007/s00018-024-05213-3 (PMC11001723; doi:10.1007/s00018-024-05213-3)
Supplement: Supplementary file 8 — (DOCX 18 KB) [file 18_2024_5213_MOESM8_ESM.docx]

**Table S1**

**Antibodies used for Western blot and Immunofluorescence analysis**

| Antibodies | Dilution | Source | manufacturer |
| --- | --- | --- | --- |
| Western blot: |  |  |  |
| anti- KIF3A | 1:1000 | Rabbit | Abclonal (A6639) |
| anti-CDK1 | 1:1000 | Mouse | Abcam (ab18) |
| anti-γ-Tubulin | 1:1000 | Rabbit | Abcam (ab179503) |
| anti-NuMA | 1:1000 | Rabbit | Abcam (ab109262) |
| anti-KIFC1 | 1:1000 | Rabbit | Biorbyt (orb101014) |
| anti-Ac-Tubulin | 1:1000 | Mouse | Sigma (T7451) |
| anti-HDAC6 | 1:1000 | Rabbit | Proteintech (16167-1-AP) |
| anti-NAT10 | 1:1000 | Rabbit | Proteintech (13365-1-AP) |
| Anti-ACTIN | 1:1000 | Mouse | Cell Signaling Technology (#3700) |
| Immunofluorescence and Immunohistochemistry: | | | |
| anti-KIF3A | 1:100 | Rabbit | Abclonal (A6639) |
| anti-KIF3A | 1:100 | Rabbit | Sangon (D220903) |
| anti-Myc | 1:100 | Mouse | Abcam (ab18185) |
| anti-Bub3 | 1:100 | Rabbit | Huabio (ET7108-82) |
| anti-γ-Tubulin | 1:100 | Rabbit | Abcam (ab179503) |
| anti-NuMA | 1:100 | Rabbit | Abcam (ab109262) |
| anti-KIFC1 | 1:100 | Rabbit | Biorbyt (orb101014) |
| anti-GM130 | 1:100 | Rabbit | Abcam (ab52649) |
| anti-Centromere | 1:100 | Human | Antibodies Incorporated (15-234) |
| anti-Ac-Tubulin | 1:200 | Mouse | Sigma (T7451) |
| anti-α-Tubulin-FITC antibody | 1:200 | Mouse | Sigma (F2168) |
| Alexa Fluor 594 Goat Anti-Rabbit IgG (H+L) | 1:200 | Goat | Abcam(ab150080) |
| Alexa Fluor 594 Goat anti-Mouse IgG (H+L) | 1:200 | Goat | Abcam(ab150116) |
| Alexa Fluor 555 Goat anti-Human IgG (H+L) | 1:200 | Goat | ThermoFisher Scientific (A-21433) |
| HRP-labeled Goat Anti-Rabbit IgG(H+L) | 1:50 | Goat | Beyotime (A0208) |
