# Supplementary material for: Kinesin KIF3A regulates meiotic progression and spindle assembly in oocyte meiosis
Source: Cell Mol Life Sci. 2024 Apr 8;81(1):168. doi: 10.1007/s00018-024-05213-3 (PMC11001723; doi:10.1007/s00018-024-05213-3)
Supplement: Supplementary file 9 — (DOCX 13 KB) [file 18_2024_5213_MOESM9_ESM.docx]

**Figure S1.** Localization of KIF3A in mouse oocytes and negative control. (A) Representative images of KIF3A localization in fixed mouse oocytes at different stages by different antibodies. Green, α‐Tubulin; red, KIF3A; blue, Hoechst, DNA. KIF3A had no specific localization in GV stage, but accumulated with microtubules in MI, ATI and MII stages. (B) Negative control for the KIF3A antibody staining in fixed mouse oocytes. IgG was adopted as the primary antibody. Green, α‐Tubulin; red, IgG; blue, Hoechst, DNA.
